# High-latitude neonate and perinate ornithopods from the mid-Cretaceous of southeastern Australia

**DOI:** 10.1038/s41598-019-56069-8

**Published:** 2019-12-20

**Authors:** Justin L. Kitchener, Nicolás E. Campione, Elizabeth T. Smith, Phil R. Bell

**Affiliations:** 1 School of Environmental and Rural Science, University of New England, Armidale, NSW 2351 Australia; 2 Australian Opal Centre, Lightning Ridge, 2834 NSW Australia

**Keywords:** Palaeontology, Palaeoecology

## Abstract

Dinosaurs were remarkably climate-tolerant, thriving from equatorial to polar latitudes. High-paleolatitude eggshells and hatchling material from the Northern Hemisphere confirms that hadrosaurid ornithopods reproduced in polar regions. Similar examples are lacking from Gondwanan landmasses. Here we describe two non-iguanodontian ornithopod femora from the Griman Creek Formation (Cenomanian) in New South Wales, Australia. These incomplete proximal femora represent the first perinatal ornithopods described from Australia, supplementing neonatal and slightly older ‘yearling’ specimens from the Aptian–Albian Eumeralla and Wonthaggi formations in Victoria. While pseudomorphic preservation obviates histological examination, anatomical and size comparisons with Victorian specimens, which underwent previous histological work, support perinatal interpretations for the Griman Creek Formation femora. Estimated femoral lengths (37 mm and 45 mm) and body masses (113–191 g and 140–236 g), together with the limited development of features in the smallest femur, suggest a possible embryonic state. Low body masses (<1 kg for ‘yearlings’ and ~20 kg at maturity) would have precluded small ornithopods from long-distance migration, even as adults, in the Griman Creek, Eumeralla, and Wonthaggi formations. Consequently, these specimens support high-latitudinal breeding in a non-iguanodontian ornithopod in eastern Gondwana during the early Late Cretaceous.

## Introduction

The discovery of dinosaurs at high paleolatitudes (>60° North or South) prompts considerable discussion on the coping mechanisms needed to accommodate the associated seasonality^1–6^. In the Northern Hemisphere, sites within the Cretaceous Arctic Circle, such as the Kakanaut Formation in north-eastern Russia (~76°N paleolatitude) and the Prince Creek Formation in northern Alaska (~85°N paleolatitude), preserve diverse dinosaur assemblages including evidence of eggs and hatchlings^7,8^. Diverse high-paleolatitude faunas are also known from the Southern Hemisphere, including Antarctica, Australia, and New Zealand^9–12^. These rich assemblages show that, unlike extant ectothermic reptiles, dinosaurs maintained a high diversity from low-to-high latitudes. Diversity peaked in temperate zones, suggesting a link to the distribution of land mass, and reduced climatic constraints^13^. Modern high-latitude vertebrates exhibit a variety of adaptive strategies to cope with seasonal change, which are broadly encompassed within the alternative overwintering strategies of migration and residency^14,15^. Although the Cretaceous climate was relatively mild by today’s standards, high-latitude dinosaurs likely employed similar adaptive strategies to deal with the seasonality and the lack of sunlight^16,17^.

In the Northern Hemisphere, evidence of nesting behavior at high-paleolatitudes reveals that dinosaurs could breed in polar regions. In north-eastern Russia, the Maastrichtian Kakanaut Formation preserves hadrosaurid and non-avian theropod eggshell fragments^8^ and, in northern North America, the Maastrichtian Prince Creek Formation, Alaska, U.S.A.^2,14,18^, and the Campanian Wapiti Formation, Alberta, Canada^19,^ preserve dental and skeletal elements of nestling-sized hadrosaurids. Examples of neonate (recently hatched) dinosaurs from the Southern Hemisphere come from more temperate paleolatitudes (<50°S)^20^ and, with the exception of a possible neonatal specimen of *Talenkauen*^21^, neonatal ornithopod material has been identified but not previously described from the Southern Hemisphere.

The high-paleolatitude (~60–70°S) mid-Cretaceous Australian dinosaur faunas of the Griman Creek, Eumeralla, and Wonthaggi formations preserve a particularly diverse array of small-bodied, bipedal non-iguanodontian ornithopods^9,12,22–24^. The diversity of these animals suggests that they were well suited to the high-latitude conditions^23,24^, although their breeding habits remain unknown, due to the absence of appropriate material.

Work on Victorian ornithopod material has previously identified juvenile specimens, although implications for high-latitude breeding were not explicitly discussed. Recent histological investigations on a large sample of Victorian material identified neonate ornithopods from the Wonthaggi Formation based on the highly vascular woven bone texture, the presence of an apparent ‘hatching’ line in one specimen (indicated by a sudden transition from fibro-lamellar to parallel-fibred bone texture), and the absence of cyclical growth marks^25,26^. A summary of small ornithopod material from the Eumeralla and Wonthaggi formations mentions a 29 mm long femur (NMV P208159)^24^. As this bone lacks definition at the proximal and distal ends, diagnostic characters are limited to the apparent proximally positioned fourth trochanter. Due to the lack of available characters, the specimen has not been described here, although we consider it plausibly represents an embryonic ornithopod femur. Despite the implications for interpreting high-latitude breeding, these previous studies presented an overview of material^24^ and focused largely on the relative growth rates of these animals rather than potential breeding strategies^25,26^.

Here, we describe two miniscule and partial ornithopod femora from the Griman Creek Formation (GCF; Cenomanian^9^) in central-northern New South Wales, Australia, and build upon previous descriptions of femora from the Eumeralla and Wonthaggi formations. Although histological analysis is obviated by the pseudomorphic opalization of some fossils from the Griman Creek Formation (opalised fossils typically do not preserve bone microstructure)^22^, gross anatomical comparisons with the histologically sampled specimens from the Eumeralla and Wonthaggi formations^25,26^ indicate that the GCF specimens represent perinate (around the point of hatching) individuals. Together, these specimens constitute the first evidence of perinatal dinosaurs from Australia and, more broadly, the first insights into the high-latitude breeding preferences of non-iguanodontian ornithopods in Gondwana. The significance of hatchling dinosaurs in Australia is discussed within the context of adaptations to high-latitude environments^2,7,19,27^.

## Localities, Geological Settings, and Paleoenvironments

**Figure 1:**
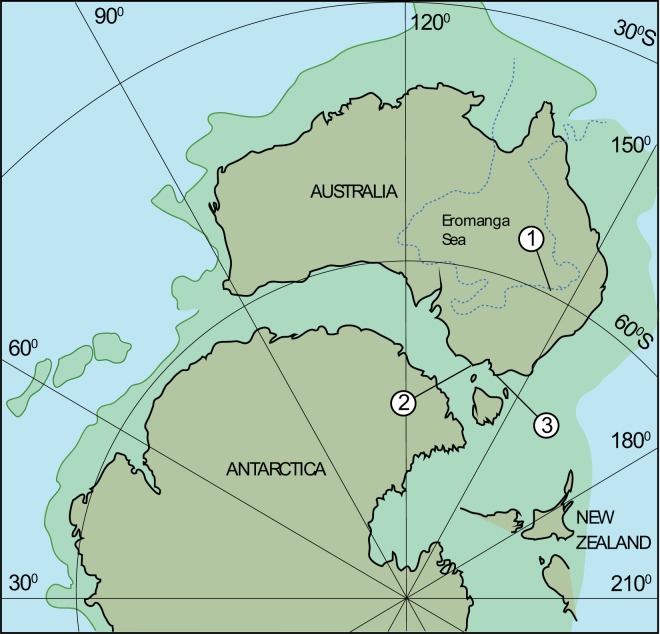
Palaeogeographic map of Australia at the Albian/Cenomanian boundary (100 Ma) showing the fossil localities discussed in this paper. (1) Lightning Ridge, Griman Creek Formation (Cenomanian); (2) Dinosaur Cove, Eumeralla Formation (Albian); (3) Flat Rocks, Wonthaggi Formation (Aptian). Although west–east rifting had commenced between Australia and Antarctica, seafloor spreading did not commence until ~94 Ma. Solid green lines represent known palaeoshorelines. Map created with Adobe Illustrator v.23.0.3 (www.adobe.com/illustrator) using palaeogeographic reconstructions generated in GPlates v.1.3.0 (www.gplates.org)^20^ with datasets from Seton *et al*.^95^. Uncertain eastern Gondwanan margin based on Milan *et al*.^96^.

The two new femora (LRF 0759 and LRF 3375) were recovered from subterranean exposures of the Griman Creek Formation (Rolling Downs Group, Surat Basin) near the town of Lightning Ridge in central-northern New South Wales, Australia (Fig. 1). Both specimens derive from laterally extensive, but discontinuous, clay-rich horizons (informally, the ‘Finch Clay’ facies) within the Wallangulla Sandstone, collectively interpreted as a lowland fluvial system punctuated by large freshwater lakes^9^. Rivers likely drained north–northeast into the epicontinental Eromanga Sea. Intermittent connections between some of these lakes and the inland sea is evinced by the rare occurrence of marine vertebrates (e.g., sharks and plesiosaurs); their rarity, however, and the occurrence of exclusively freshwater invertebrate taxa (e.g., gastropods and unionid bivalves) indicate that such marine connections lay distal to the study area at Lightning Ridge^9^. Recent detrital zircon analyses constrained the minimum depositional age of the Griman Creek Formation at Lightning Ridge to the early to mid-Cenomanian (100.2–96.6 Ma)^9^. During the Cenomanian, the area was located at a paleolatitude of ~60°S^20^, approaching the paleoantarctic circle. The paleoclimate at these latitudes was one of high precipitation (humid–perihumid)^28^ and mild temperatures, given the diversity of Testudines and Crocodylomorpha^29–32^. The area possibly had a mean annual average temperature (MAAT) of ~14 °C, based on the minimum thermal tolerance of modern crocodylians^33^.

The specimens from the Eumeralla Formation (Aptian–Albian, Otway Group; NMV P186004, NMV P198900) come from the Dinosaur Cove locality, Otway Basin, Victoria, Australia. Recent fission track-calibrated biostratigraphic work places this locality within the early Albian *Crybelosporites striatus* Zone^34^. Specimens from the slightly older Wonthaggi Formation (Barremian–Aptian, upper Strzelecki Group; NMV P198982, NMV P216768) derive from the Flat Rocks locality, Gippsland Basin, Victoria, Australia, which has been assigned to the late Aptian upper *Cyclosporites hughesii* Zone^35^. Both localities were deposited at a paleolatitude of ~70°S^20^ within extensional terrains of the Australian-Antarctic rift valley. Paleoenvironmental reconstructions suggest floodplains, freshwater lakes, and braided streams dominated the valley lowlands^12,36–38^. The paleoclimate in this region was wet (humid–perihumid), highly seasonal and potentially much cooler (MAAT = −6 to +10 °C)^36^ than that of the Griman Creek Formation. A cold paleoclimate, including winter freezing, is supported by extremely low δ^18^O values of meteoric fluids, the presence of possible cryoturbation structures (earthy hummocks and involutions), and the leaf size and physiognomy of local flora^28,36,39–42^.

### Institutional abbreviations

AM, Australian Museum, Sydney, New South Wales, Australia, LRF, Australian Opal Centre, Lightning Ridge, New South Wales, Australia, MV, Museums Victoria, Melbourne, Victoria, Australia (formerly, National Museum of Victoria (NMV)).

## Methods

Previous histological work on a subset of nine ornithopod femora from the Eumeralla and Wonthaggi formations documented between 0 and 8 cyclical growth marks (CGMs), regarded as a record of annual growth^43,44^. In addition, the presence of an external fundamental system (EFS) in some of the largest specimens marks the cessation of appreciable growth and the onset of skeletal maturity^25^. The smallest of these femora (NMV P216768; length≈48 mm) lacks CGMs and displays an abrupt transition from fibro-lamellar to parallel-fibred tissues hypothesized to be a ‘hatching line’^25^, similar to that seen in a neonate sauropod from Madagascar^45^. A ‘hatching line’ is a distinct transition of bone texture observed at the point of hatching in squamates, crocodilians, and some birds, and the point of birth in some mammals. As a result, the size of NMV P216768 was chosen as arbiter for femora of animals zero to one year old and a threshold of < 60 mm was proposed for femoral length^25^.

### Femur length estimation

The lengths of the incomplete proximal femora (LRF 0759, LRF 3375) were estimated via an ordinary least squares (OLS) regression model implemented in the statistical software R v. 3.5.3^46^. The OLS model compares anteroposterior width at the midpoint to overall length and was derived from measurements of 16 Australian small ornithopod femora (Table 1). Measurements were taken directly from specimens using digital calipers. Dimensions of specimens not measured directly were taken from prior studies^25^ or measured from published images^24,47,48^ using ImageJ v. 1.8.0^48^. The femoral measurements were not log transformed, as it was noted that the distribution of residuals about the regression line could not be differentiated from a normal distribution (see Supplementary Information). Uncertainty in femoral length estimates was measured as the 95% prediction interval about the regression line as it more accurately describes the spread of the data.

**Table 1:** Measurements of femora examined in this study, with additional specimens from Rich and Vickers-Rich^24^, Herne^47^ and Woodward *et al*.^25^. ^D^ML and ^D^AP refer to mediolateral and anteroposterior diameters, respectively. Circumferences in brackets (i.e. [x]) were calculated from the ^D^ML and ^D^AP measurements.

Specimen	Locality	Formation	Element	Length (mm)	^D^ML (mm)	^D^AP (mm)	Circumference (mm)	Source of data
LRF 759	Lightning Ridge	Griman Creek	Proximal left femur	—	3.73	4.06	[12.24]	This study
LRF 3330	Lightning Ridge	Griman Creek	Proximal left femur	—	—	4.39	[13.79]	This study
F105673	Lightning Ridge	Griman Creek	Incomplete left femur	—	9.8	12.5	[35.16]	This study
F127930	Lightning Ridge	Griman Creek	Incomplete left femur	—	12.76	15.54	[44.56]	This study
NMV P186004	Dinosaur Cove	Eumeralla	Left femur	47.07	5.75	4.65	[16.38]	This study
NMV P198900	Dinosaur Cove	Eumeralla	Left femur	54.7	6.26	3.7	[15.90]	This study
NMV P198982	Flat Rocks	Wonthaggi	Right femur	60.7	6	6.85	[20.21]	This study
NMV P216768	Flat Rocks	Wonthaggi	Right femur	47.85	5.15	6.46	[18.30]	This study
NMV P179561	Dinosaur Cove	Eumeralla	Right femur	68.93	4.65	9.92	[23.64]	This study
NMV P185980	Dinosaur Cove	Eumeralla	Left femur	58.95	6.16	7.81	[22.02]	This study
NMV P186326	Dinosaur Cove	Eumeralla	Left femur	185	19	20.68	[62.36]	This study
NMV P185999	Dinosaur Cove	Eumeralla	Femur	95	—	11.5	—	Rich & Vickers-Rich^24^
NMV P208186	—	Wonthaggi	Femur	189	—	23	—	Rich & Vickers-Rich^24^
NMV P199146	Flat Rocks	Wonthaggi	Femur	174	—	20	—	Rich & Vickers-Rich^24^
NMV P186468	Flat Rocks	Wonthaggi	Femur	128	—	16	—	Rich & Vickers-Rich^24^
NMV P198888	Flat Rocks	Wonthaggi	Femur	102	—	13	—	Rich & Vickers-Rich^24^
NMV P186403	Eagle’s Nest	Wonthaggi	Femur	110	—	14	—	Rich & Vickers-Rich^24^
NMV P198963	Flat Rocks	Wonthaggi	Femur	138	—	17	—	Rich & Vickers-Rich^24^
NMV P186370	Flat Rocks	Wonthaggi	Femur	130	—	17	—	Rich & Vickers-Rich^24^
NMV P186047	Dinosaur Cove	Eumeralla	Femur	134	—	17	—	Herne^47^
NMV P177935	Dinosaur Cove	Eumeralla	Femur	208	—	—	75	Woodward^25^
NMV P221151	Flat Rocks	Wonthaggi	Femur	160	—	—	55	Woodward^25^

### Body mass estimation

Body mass was estimated using the R package MASSTIMATE v. 1.4^49,50^, which now incorporates the developmental mass extrapolation (DME) approach based on Erickson and Tumanova^51^ (see Supplementary Information for R script). DME is the most appropriate mass estimation approach for juveniles as it cannot be assumed that intraspecific growth-related patterns will follow adult-based interspecific circumference–mass relationships such as those originally proposed by Anderson *et al*.^52^. To conduct the DME, first the mass of an adult representative (*BM*_*adult*_) was calculated using its minimum circumference and the bipedal corrected equation from Campione *et al*.^50^. This value was then scaled based on the proportion between the cube of the juvenile femoral lengths (*FL*_*juvenile*_^3^) and the cube of the adult femoral length (*FL*_*adult*_^3^):

$$B{M}_{juvenile}=B{M}_{adult}\times \frac{F{{L}_{juvenile}}^{3}}{F{{L}_{adult}}^{3}}$$

NMV 177935 was chosen as the adult proxy for the DME (*BM*_*adult*_) as it is the largest femur (208 mm) found to display an external fundamental system^25^.

### Age estimation

The ontogenetic stage or age of fossil vertebrates is usually investigated histologically^53^. Periodic variation in bone textures observed in histological analyses can indicate seasonal growth and cyclical growth marks (CGMs), which can act as a proxy for the individual’s age. For this approach to be viable, the microtexture of the target bone must be well preserved. In instances of pseudomorphic preservation, such as the opalized Griman Creek Formation specimens in this study, bone microtexture is lost in preservation and, therefore, alternatives to histology must be employed.

In order to estimate the ages of the Griman Creek Formation ornithopod femora, we first explored the relationships between age and circular growth mark (CGM) circumference in Australian small ornithopod femora published in the supplementary information of Woodward *et al*.^25^. Three specimens with multiple CGMs (NMV P221151, NMV P186326, NMV P177935) and two smaller specimens (NMV P216768, NMV P208495) were included. Both NMV P221151 and NMV P186326 followed an evident linear pattern and were thus expressed as an OLS model, whereas NMV P177935 follows a non-linear power function and was modeled via a nonlinear least squares (NLS) model.

To maximize the sample used for age estimation, an overall OLS linear regression was generated between age and CGM circumferences in all available specimens (Table 1). The validity of this overall estimation model was evaluated by calculating its 95% prediction errors, which were compared to the OLS and NLS models derived from single specimens. Ages for the focal specimens (LRF 0759, LRF 3375) were predicted based on their surface circumferences, working on the assumption that CGMs represent the surface circumferential dimensions of the bone at the point in time when they were created^54,55^. While the CGM circumferences of specimens from Woodward *et al*.^25^ were measured digitally from thin section images, the dimensions of our focal specimens were measured by hand; circumferences of these femora were calculated as ellipses using mediolateral and anteroposterior diameters as the elliptical axes. Diameters were taken with digital calipers at the narrowest available section of the femur. Ellipse circumference was approximated using one of the Ramanujan formulations based on *h*,

$$h=\frac{{(a-b)}^{2}}{{(a+b)}^{2}}$$

where *a* is half of the anteroposterior diameter and *b* is half of the mediolateral diameter. The circumference (C) can then be approximated through,

$$C\approx \pi (a+b)(1+\frac{3h}{10+\sqrt{4-3h}})$$

Due to the preservation of LRF 3375, only the anteroposterior diameter could be taken and thus a circular circumference was calculated.

## Results

### Description of material from the Griman Creek Formation, New South Wales

**Figure 2:**
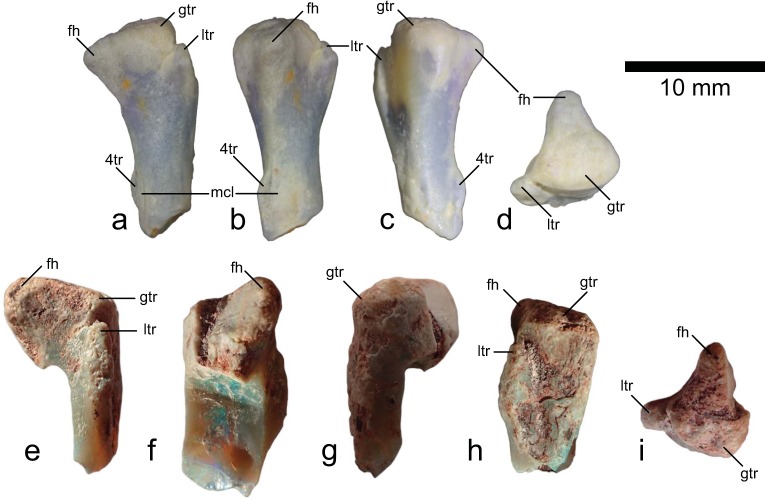
Proximal parts of ornithopod femora from the Griman Creek Formation. LRF 0759 (**a**–**d**). LRF 3375 (**e**–**i**). (**a,e**) Anterior views; (**b,f**) medial views; (**c,g**) posterior views; (**d,i**) proximal views; (**h**) lateral view. Abbreviations: 4tr, fourth trochanter; fh, femoral head; gtr, greater trochanter; ltr, lesser trochanter; mcl, *M. caudofemoralis longus* insertion scar.

LRF 0759 (maximum length = 16 mm) and LRF 3375 (maximum length = 16 mm) are partial proximal right femora. LRF 0759 is broken just below the fourth trochanter (Fig. 2), whereas LRF 3375 is broken obliquely just below the base of the lesser trochanter so that the medial portion of the femoral shaft is also missing (Fig. 2b). The two specimens differ noticeably in the form and orientation of the femoral head. In LRF 0759, the femoral head is rounded-triangular in anterior and proximal views and is angled, ~65°, to the diaphysis (Fig. 2), resulting in a ventral position for the femoral head relative to the greater trochanter. In LRF 3375, the femoral head is more ‘tongue’-shaped in anterior view and forms an angle of ~110° to the diaphysis (Fig. 2). The femoral head in LRF 3375 is sheared in a vertical plane, but terminates dorsal to the greater trochanter. Other features in both specimens are similar. A distinct *fossa trochanteris* is absent. The lesser trochanter is significantly lower than the greater trochanter and is separated from it by a shallow cleft (Fig. 2). The lateral contour of the greater trochanter is gently convex in proximal view. The preserved portion of the femoral diaphysis is straight in lateral and medial views and the low, arcuate fourth trochanter (present only on LRF 0759) appears to be within the proximal half of the femur (Fig. 2). A broad depression on the medial surface of the femoral diaphysis, adjacent to the fourth trochanter, marks the insertion site for the *M. caudofemoralis longus* (Fig. 2).

### Description of material from the Eumeralla Formation, Victoria

**Figure 3:**
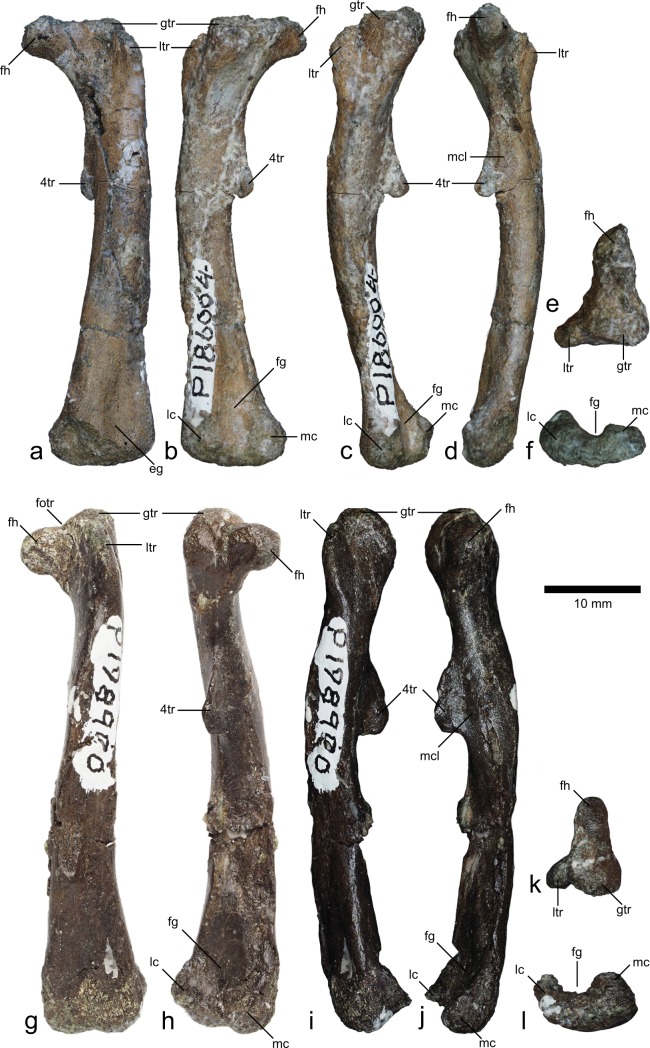
Ornithopod femora from the Eumeralla Formation. NMV P186004 (**a–f**). NMV P198900 (**g–l**). (**a,g**) Anterior views; (**b,h**) posterior views; (**c,i**) lateral views; (**d,j**) medial views; (**e,k**) proximal views; (**f,l**) distal views. Abbreviations: 4tr, fourth trochanter; eg, extensor fossa; fg, flexor fossa; fh, femoral head; fotr, *fossa trochanteris*; gtr, greater trochanter; lc, lateral condyle; ltr, lesser trochanter; mc, medial condyle; mcl, *M. caudofemoralis longus* insertion scar.

NMV P186004 (length = 47 mm) and NMV P198900 (length = 55 mm) are complete, well-preserved left femora (Table 2; Fig. 3). Both femora are similar in most respects, except for the shape of the proximal end. In NMV P186004, the femoral head is angled approximately 90° to the diaphysis and the femoral head appears to be at the same level as the greater trochanter, although damage to the proximal end makes this uncertain (Fig. 3). By contrast, the femoral head of NMV P198900 is angled at approximately 80° to the diaphysis, the relatively low angle accentuated by the rather sinuous profile of the femur in anterior view (Fig. 3). As a result, the greater trochanter is higher than the femoral head (Fig. 3). In NMV P198900, the femoral head and greater trochanter are separated by a shallow, saddle-shaped *fossa trochanteris* (Fig. 3). The form of the *fossa trochanteris* in NMV P186004 is unclear due to damage to the proximal end of the femur. The lesser trochanter terminates below the level of the greater trochanter and the two are separated from each other by a short, deep cleft. The femoral diaphyses are bowed in lateral view, although NMV P198900 displays a sigmoidal (rather than straight) anterior profile owing to the medial tilt of the proximal end. The fourth trochanter is large and pendant (but missing the distal extremity) and within the proximal half of the femur. The *M. caudofemoralis longus* insertion scar forms a distinct depression on the medial surface of the femoral shaft, adjacent to the fourth trochanter. The distal end is mediolaterally expanded to form the medial and lateral condyles. In NMV P198900 the medial condyle is the larger of the two, whereas the opposite is true for NMV P186004. The lateral margin of the lateral condyle is convex in distal aspect (i.e. a medially inset lateral condylid is absent). The distal flexor fossa is broad and U-shaped in distal view (Fig. 3), whereas the extensor fossa on the anterior surface of the femur is weakly developed and not visible in distal aspect (Fig. 3).

**Table 2:** Morphotype designations for femora examined in this study.

Specimen	Locality	Formation	Element	Inferred developmental stage	Previous assignment(s)	Current designation
LRF 759	Lightning Ridge	Griman Creek	Proximal left femur	Embryonic	—	Morphotype 1
LRF 3330	Lightning Ridge	Griman Creek	Proximal left femur	Perinate	—	Morphotype 2
NMV P186004	Dinosaur Cove	Eumeralla	Left femur	Neonate	*Leaellynasaura amicagraphica*^b^*;* Victorian Ornithopod Postcranium Type I (VOCP I) morphotype^c^	Morphotype 3
NMV P198900	Dinosaur Cove	Eumeralla	Left femur	Neonate	*Leaellynasaura amicagraphica*^a^	Morphotype 3
NMV P198982	Flat Rocks	Wonthaggi	Right femur	‘Yearling’	*Fulgurotherium australis*^b^	Morphotype 4
NMV P216768	Flat Rocks	Wonthaggi	Right femur	Neonate^a^	—	Morphotype 4?

^a^Woodward *et al*.^25,26^; ^b^Rich and Vickers-Rich^24^; ^c^Herne^47^.

### Description of material from the Wonthaggi Formation, Victoria

**Figure 4:**
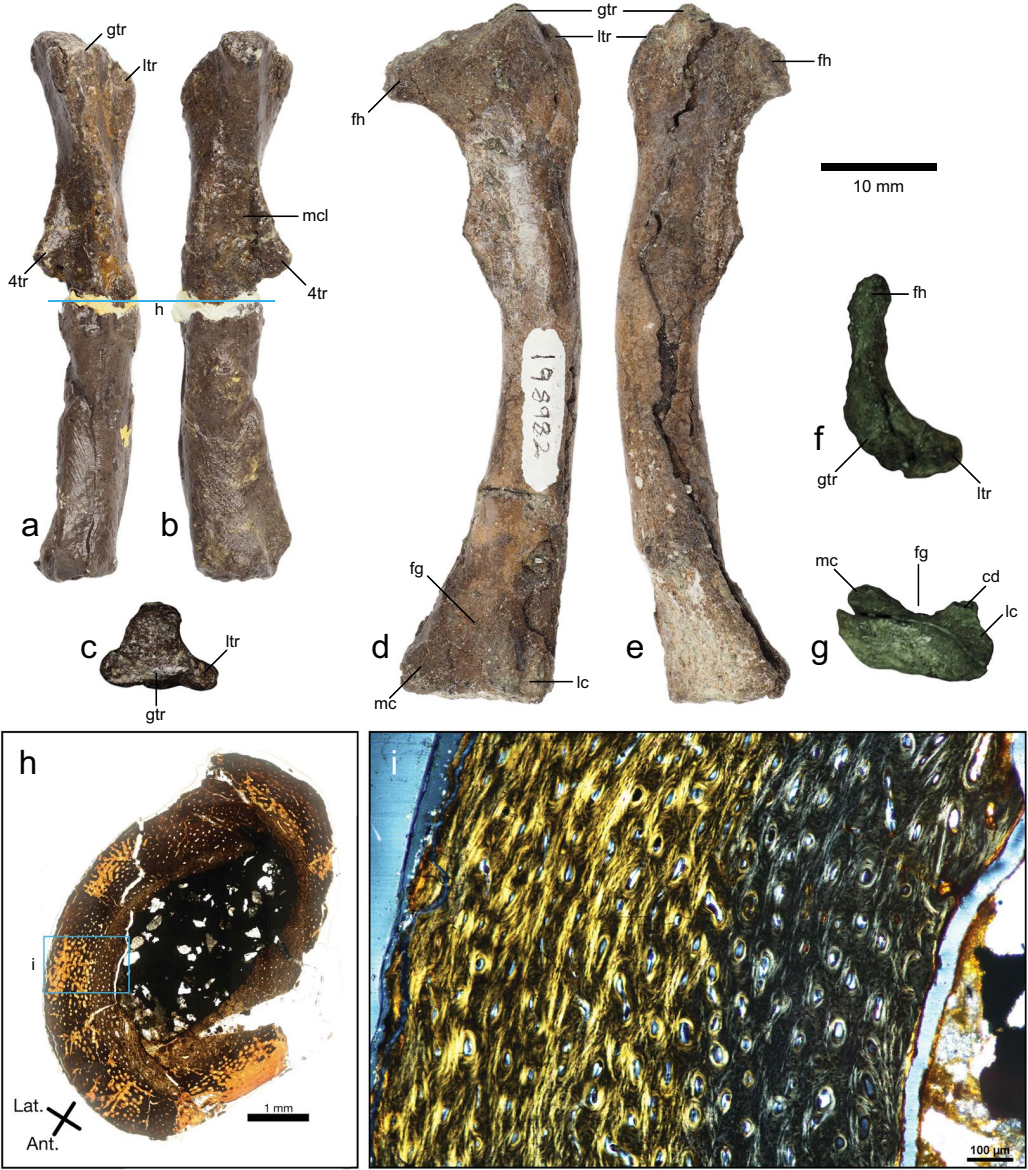
Ornithopod femora from the Wonthaggi Formation. NMV P216768 (**a–c,h,i**). NMV P198982 (**d–g**). (**a**) Lateral view; (**b**) medial view; (**c,f**) proximal views; (**d**) posterior view; (**e**) anterior view; (**g**) distal view. (**h,i**) Thin section of NMV P216768 from Woodward *et al*.^25^. (**h**) Complete thin section in plane polarized light. (**i**) Close up of area from (**h**) defined by blue rectangle in circularly polarized light. ‘Hatching line’ in (**i**) visible as change in color and related to the transition from a fibro-lamellar to a poorly organized parallel-fibred texture^25^. Location of thin section indicated by blue lines across (**a**) and (**b**). Abbreviations: 4tr, fourth trochanter; cd, lateral condylid; fg, flexor fossa; fh, femoral head; gtr, greater trochanter; lc, lateral condyle; ltr, lesser trochanter; mc, medial condyle; mcl, *M. caudofemoralis longus* insertion scar.

NMV P198982 (length = 61 mm) and NMV P216768 (length = 48 mm) are nearly complete right femora lacking their distal articular ends (Fig. 4). NMV P198982 is diagenetically compressed anteroposteriorly, particularly at the proximal end. As a result, the femoral head appears bladelike in proximal view and oriented at approximately 80° to the diaphysis (in anterior view). The greater trochanter is higher than the femoral head proximally, but distortion and damage on both features obscures the transition between them (Fig. 4). In NMV P216768, damage to the proximal end renders the form and orientation of the femoral head and the form of the *fossa trochanteris* unclear. The proximal end of the lesser trochanter is lower than the greater trochanter, with a shallow cleft between them. The lateral contour of the greater trochanter is flat in NMV P216768 (in proximal aspect) but cannot be accurately observed in NMV P198982 due to distortion.

In both specimens, the femoral diaphysis is straight in anterior view and gently bowed in lateral view. The fourth trochanter is proximally positioned but incomplete in both specimens. A depression for the *M. caudofemoralis longus* is found medially, immediately adjacent to the base of the fourth trochanter. In NMV P198982, where the distal end is partially preserved, the flexor fossa forms a broad, open ‘U’ in distal aspect. A distinct lateral condylid is present, and is medially inset from the lateral edge of the lateral condyle (Fig. 4). The extensor fossa is shallow and not visible in distal aspect. The corresponding distal portion is missing in NMV P216768.

### Femur length, body mass, and age estimates

**Figure 5:**
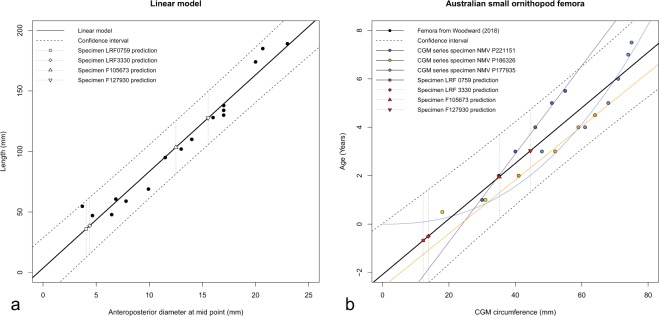
Plots of scaling models. (**a**) Linear regression model of femur length predicted by anteroposterior diameter at the femoral mid-point. (**b**) Linear regression model of individual age predicted by circular growth mark circumference sequences. CGM circumferences from supplementary data published with Woodward *et al*.^25^.

The reconstructed lengths of the two Griman Creek Formation specimens, LRF 0759 and LRF 3375, were estimated at 36 mm (upper maximum value of 59 mm) and 39 mm (upper maximum value of 62 mm) long, respectively (Table 3; Fig. 5a). Length estimates for two larger incomplete small-ornithopod femora from the Griman Creek Formation (AM F105673, AM F127930) were also included (Fig. 5a). These were 82–125 mm and 106–150 mm long, respectively: substantially larger than the focal specimens. Mass estimates, calculated via DME, indicate that LRF 0759 and LRF 3375 represent the smallest individuals thus far sampled. Based on the point femoral length estimate and the ~25% mean per cent prediction error of the adult proxy, the body mass of LRF 0759 was likely between 113 and 191 g and that of LRF 3375 was likely between 140 and 236 g. Mass estimates for the juveniles from the Eumeralla and Wonthaggi formations ranged from 251–424 g for the smallest (NMV P186004) to 788–1332 g for the largest (NMV P179561) (Table 3).

**Table 3:** Length and mass estimates for small Australian ornithopod femora. Incomplete lengths estimated via linear regression. Masses estimated with MASSTIMATE^49,50^ and developmental mass extrapolation (DME), based on Erickson and Tumanova^51^. Ages estimated via linear regression. Estimates expressed as point estimates followed by 95% prediction intervals in parentheses.

Specimen	Locality	Formation	Element	Length (mm)	Estimated Length (mm)	DME Estimated Mass (g)	Estimated age (years)
LRF 759	Lightning Ridge	Griman Creek	Proximal left femur	16.05 (incomplete)	36.08 (12.67–59.48)	151.96 (113.02–190.91)	−0.68 (−2.62–1.27)
LRF 3330	Lightning Ridge	Griman Creek	Proximal left femur	15.8 (incomplete)	38.71 (15.41–62.01)	187.73 (139.62–235.85)	−0.50 (−2.42–1.43)
F105673	Lightning Ridge	Griman Creek	Incomplete left femur	108.45 (incomplete)	103.43 (81.5–125.35)	3581.01 (2663.19–4498.82)	1.95 (0.21–3.69)
F127930	Lightning Ridge	Griman Creek	Incomplete left femur	75.04 (incomplete)	127.68 (105.65–149.72)	6738.08 (5011.11–8465.06)	3.03 (1.33–4.74)
NMV P186004	Dinosaur Cove	Eumeralla	Left femur	47.07	—	337.56 (251.04–424.08)	—
NMV P198900	Dinosaur Cove	Eumeralla	Left femur	54.7	—	529.76 (393.98–665.54)	—
NMV P198982	Flat Rocks	Wonthaggi	Right femur	60.7	—	723.91 (538.37–909.45)	—
NMV P216768	Flat Rocks	Wonthaggi	Right femur	47.85	—	354.62 (263.73–445.51)	—
NMV P179561	Dinosaur Cove	Eumeralla	Right femur	68.93	—	1060.09 (788.39–1331.8)	—
NMV P185980	Dinosaur Cove	Eumeralla	Left femur	58.95	—	663.09 (493.14–833.04)	—
NMV P186326	Dinosaur Cove	Eumeralla	Left femur	185	—	20494.36 (15241.64–25747.08)	—

Exploration of growth-related changes between CGM circumferences and ages reveal substantial differential scaling among the three NMV specimens (Fig. 5b). Nevertheless, at the size of the focal Griman Creek Formation specimens, the three models occur within the 95% prediction intervals of the overall OLS model supporting its use as a general age-estimation model. Given the overall OLS model, the ages of LRF 0759 and LRF 3375 are estimated at –0.68 years (–2.62 to 1.27 based on the 95% prediction error) and –0.50 years (–2.42 to 1.43), respectively (Fig. 5b). The complete list of length, mass, and age estimates is presented in Table 3.

## Discussion

### Taxonomic considerations

Early work on the ornithopod femora from the Eumeralla and Wonthaggi formations indicated as many as four morphotypes^23^, although this was later revised to two^24^. Nevertheless, four taxa have been named from these deposits: *Leaellynasaura amicagraphica, Qantassaurus intrepidus, Atlascopcosaurus loadsi*, and *Diluvicursor pickeringi*^23,24,37^. Molnar and Galton^56^ identified two femoral morphotypes from the Griman Creek Formation and craniodental remains suggest that up to three small-bodied non-iguanodontian ornithopods were present^22^. Larger-bodied iguanodontians were also present in the Griman Creek Formation but are absent from the Eumeralla and Wonthaggi formations^9,22,57^. Our results support the occurrence of at least two morphotypes in the Griman Creek Formation based on differences in the inclination of the femoral head (Fig. 2). Two morphotypes are also collectively recognized from the Wonthaggi and Eumeralla formations, based on the presence/absence of a medially inset lateral condylid (Table 2). However, these morphological distinctions are used merely for convenience, as femoral head inclination and the presence/absence of a lateral condylid are variably represented within the sample making the taxonomic definition of these morphotypes highly ambiguous. The pervasiveness of diagenetic alteration among the specimens from the Wonthaggi and Eumeralla formations similarly renders any taxonomic conclusions, based on these divisions, dubious^24^. Nevertheless, such morphological variations imply that several taxa are likely represented by the remains.

Characters of femora described in this study differ from those of sauropods and theropods. The well-defined femoral head and lesser trochanter are unlike those of many sauropods and thyreophorans, including known juvenile specimens, where both features are relatively ill-defined (e.g., Burns *et al*.^58^). The tongue-shaped lesser trochanter, which is separated by a narrow cleft from the greater trochanter, is unlike that in theropods (e.g., Griffin^59^; Hutchinson^60^). Other theropod (and saurischian) synapomorphies, such as a distinct fovea capitis, a deep sulcus for the ischiofemoral ligament^61^, a dorsolateral trochanter, and a trochanteric shelf, are also absent, although the absence of the latter two may be ontogenetically variable among certain theropods^59^. Although finer taxonomic assessments are impossible, all femora described in this study can be constrained to Ornithopoda based on: a bowed femoral shaft, a proximally positioned and pendant-shaped fourth trochanter, a shallow (or absent) distal extensor fossa, and an ‘open’ distal flexor fossa (e.g., Norman *et al*.^62^; Rich and Rich^23^; Rich and Vickers-Rich^24^), although not all of these features are preserved in each specimen due to the variable degrees of completeness. The two smallest specimens (LRF 0759, LRF 3375), which do not preserve most of these features because of their incompleteness and/or inferred juvenile state, possess additional characters widespread among non-iguanodontian ornithopods, including: a proximally positioned fourth trochanter and a finger-like (not blade-like) lesser trochanter that is closely appressed (rather than widely separated) to the greater trochanter. In these specimens, the straight proximal margin between the caput and trochanteric ends of the proximal femur (i.e. absence of a *fossa trochanteris*, the presence of which is a typical cerapodan feature^63^) is interpreted to be the result of the early developmental stage represented by these individuals.

### Ontogenetic interpretations

Due to the absence of bone microtexture within our focal Griman Creek Formation specimens, age estimations are based on comparisons with other Australian small ornithopod femora that were histological investigated by Woodward *et al*.^25^. Woodward and colleagues found that the growth sequences of Eumeralla- and Wonthaggi-formation small ornithopod femora displayed weakly asymptotic trends, with the annual increase in CGM circumference appearing to plateau as maturity was reached. Bone texture also reflected this growth pattern, with a shift from predominantly fibro-lamellar and poorly organized parallel fibers to a primarily parallel-fibred texture after the third CGM. Overall, there was little variation in growth rate between specimens, with the exception of NMV P180892. This much larger specimen was included to test the hypothesis that the smaller examples were in fact juveniles of a larger form. However, the presence of small femora from apparently mature individuals (exhibiting an EFS; maximum length 208 mm) within the sample, and the fact that this larger specimen was yet to reach maturity in spite of its large size (lacking an EFS at an estimated length of 315 mm), indicates that this taxon could be distinguished from the rest of the sample for its differential growth regime. With such small specimens exhibiting an EFS, the Australian small ornithopods studied in Woodward *et al*.^25^ appear to have been diminutive, even as adults.

The smallest femora within our sample are inferred to be from either neonate (NMV P216768, NMV P186004) or perinate (LRF 3375, LRF 0759) individuals based on their overall size and the presence of an apparent ‘hatching line’ in NMV P216768^25^. Age estimates from the regression based on CGM circumferences (Fig. 5b; Table 3) further support this conclusion. The smallest known Victorian specimen (MNV P208159) reported by Rich and Vickers-Rich^24^ presumably also represents a perinate. As the two Griman Creek Formation specimens (LRF 3375, LRF 0759) were incomplete, the diameter measurements are likely to exceed the true narrowest section of the femur. Therefore, point length, mass, and age estimates may well represent overestimates. In the absence of direct histological evidence, femora larger than NMV P216768, but less than 60 mm in length, were considered to be between neonate and one year old, informally referred to here as a ‘yearling’. Mass estimates for presumed perinates are around half that of the neonate NMV P216768, which further support this identification (Table 3). Although slightly larger than these specimens, the body mass of NMV P198900 overlaps with the range of other neonates (NMV P216768, NMV P186004) and may represent a third neonatal specimen (Table 3). The largest of the juvenile femora, NMV P198982, may also represent an individual less than one year old, here referred to as a ‘yearling’.

Although missing its distal half, the perinate femur LRF 0759 has a low, crescentic fourth trochanter and is unlike the typical pendant-shaped trochanter seen in more mature non-iguanodontian ornithopod individuals^62,64^ including the neonate NMV P216768. Notably, this feature is similar to the low, triangular fourth trochanter typically seen in Hadrosauroidea and some non-hadrosauroid iguanodontians^64–67^. A low, triangular fourth trochanter is also present in perinatal *Hypacrosaurus stebingeri*^68^ but was apparently absent in perinatal *Saurolophus angustirostris*^69^. The similarity in fourth trochanter morphology between the Griman Creek Formation specimens and most iguanodontians hints at a possible heterochronic shift in the evolutionary history of ornithopods, in which iguanodontians paedomorphically (specifically via neoteny, reduction in developmental rate) retain juvenile characteristics into adulthood. Independent of the functional nature of the pendant fourth trochanter in adult non-iguanodontian ornithopods^64^, a hypothesized association between the loss of this feature and quadrupedality in ornithischians (*sensu* Maidment and Barrett^70^) presents the intriguing possibility that the evolution of quadrupedality results from such heterochronic transitions, at least in ornithopods. Similar shifts, associated with limb proportions, have been proposed for the evolution of quadrupedality in sauropods^71,72^. This hypothesis suggests that juvenile ornithopods are, if anything, less bipedal than their adult counterparts (but see Dilkes^73^) or, at least, limited in their degree of function at these early stages of development. Limited functionality is supported by the absence of a distinct *fossa trochanteris* in LRF 0759, LRF 3375, and NMV P198982 and the absence of insertion scars for the *M. iliotrochantericus* on the posterior surface of the femoral head (except for NMV P198900 and NMV P186004) and the *M. caudofemoralis brevis* on the fourth trochanter. Their absence or limited development in the present material implies the gradual acquisition of these traits through repetitive muscle action as the individual grew^74^.

### Ornithopod nesting environments at high palaeolatitudes

The presence of perinate and neonate ornithopods from high-palaeolatitudes in south-eastern Australia invites palaeoenvironmental comparisons with similar nesting sites from the Northern Hemisphere. To date, evidence of ornithopod breeding at high-palaeolatitudes in the Northern Nemisphere is limited to hadrosaurid remains discovered in U.S.A., Canada, and Russia. The late Maastrichtian Kakanaut Formation (70–75°N palaeolatitude)^75^ in north-eastern Russia produces hadrosaurid and other non-avian dinosaur eggshell fragments^8^. A low-energy lowland depositional environment was inferred from fine-grained sediments and the presence of fish remains within the fossiliferous lens. Ectothermic tetrapods, such as crocodilians and turtles, appear to have been absent. In the Campanian units of the Wapiti Formation (western Canada; ~65°N palaeolatitude), hadrosaurid nesting is evinced by hatchling-sized skeletal elements^19^. The palaeoenvironmental setting was interpreted as a low-energy fluvial deposit, punctuated by floods and coal-forming wetlands^19,76^. Squamates and turtles were present, perhaps reflecting a milder climate as resulting from the proximity to the Western Interior Seaway. The most northerly evidence of dinosaur breeding comes from the Maastrichtian Prince Creek Formation in northern Alaska, U.S.A. (~85°N palaeolatitude)^7^. Hatchling-sized hadrosaurids, along with small-bodied non-iguanodontian ornithopods and dromaeosaurid teeth, were recovered from channel-lag, overbank, and pond deposits formed in lowland riverine, floodplain, and deltaic environments^77–79^. Amphibians and reptiles (excepting dinosaurs) were absent, although a partial turtle carapace was recovered from earlier, Cenomanian deposits on the Alaskan North Slope^7,79,80^.

High-latitude ornithopod breeding sites in the Northern Hemisphere were located in lowland settings whereas, at lower palaeolatitudes, nesting sites occupied both dry upland and wet lowlands regions^19,68,81^. The variation between high and low latitude nesting environments suggests the possibility of latitudinally driven nesting strategies among hadrosaurid ornithopods. Whether such latitude-dependent strategies occur in non-iguanodontian ornithopods, however, remains unknown. Neonate tooth crowns attributed to the elasmarian *Talenkauen santacrucensis*^21^ constitute the sole record of a ‘nestling’ non-iguanodontian ornithopod outside of Australia. These teeth derive from the Campanian–Maastrichtian Cerro Fortaleza Formation in southern Argentina, which was variably deposited in fluvial, fluvial-palustrine, and coastal floodplain environments^82–84^. Palaeogeographic reconstructions place southern Argentina at ~50°S^20^, considerably more equatorial than the Australian localities studied herein.

In Australia, the Wallangulla Sandstone (Griman Creek Formation) is interpreted as a lowland, near-coastal palaeoenvironment dominated by freshwater lakes and streams and at a palaeolatitude of ~60°S^9,20,85^. The presence of diverse ectothermic tetrapods, such as squamates, turtles, and crocodylomorphs, indicates a milder climate than some of the higher-palaeolatitude sites, including the Eumeralla and Wonthaggi formations^9,29–32,86^. The Eumeralla and Wonthaggi formations were deposited within the Australian-Antarctic rift valley at a palaeolatitude of ~70°S^38,87^ and were interpreted as representing wet lowlands, large braided rivers, forested floodplains, and shallow lakes^37,38^. Palaeontological, palaeobotanical, sedimentological, and isotopic data suggest cold mean annual temperatures, between –6 to +5 °C, but mean annual temperatures as high as 10 °C were also proposed^28,36,39–41^. Furthermore, cooler climates for the Eumeralla and Wonthaggi formations, compared to the more northerly Griman Creek Formation, are supported by a depauperate mesoreptile fauna^9,41^.

In general, the nesting sites of high-palaeolatitude ornithopods, including hadrosaurids from the Northern Hemisphere and non-iguanodontian ornithopods from the Southern Hemisphere, were restricted to moist, lowland settings. This limited record is undoubtedly biased, not least by the Australian fossil record, where appropriate strata—both in terms of age and depositional setting—are constrained to a handful of localities. Nevertheless, if the apparent association between ornithopod nesting sites and lowland environments truly reflects ornithopod nesting choices at high latitudes, it may be explained by temperature constraints on egg incubation. In oviraptorosaurs, estimates suggest bird-like incubation temperatures (~35–40 °C)^88^. Incubation temperatures have not been estimated for ornithopods, however, the porosity of their eggshells suggests the use of covered nests buried in organic matter, where bacterial respiration can provide the main source of heat^89^. The necessity for a stable nest microclimate for incubation purposes may have limited high-latitude ornithopods to breeding in lowland environments, which would have generally been warmer and less prone to extreme temperature fluctuations than upland settings^90^.

### Implications for overwintering strategies

Migration was proposed as an overwintering strategy for high-palaeolatitude dinosaurs, although current evidence contends that most were year-round residents^1,2,8,14^. High-latitude zones receive above-average sunlight and can be highly productive during the summer, making these regions attractive breeding destinations for migratory animals and perennial residents. Migrators that breed at high latitudes are fast and efficient travelling animals, but migratory strategies are contingent on precocial young capable of joining their parents on the journey to lower latitudes prior to the winter season. Such migratory strategies are particularly prevalent among birds, which are able to travel rapidly and efficiently *via* flight^91^. In contrast, migration is rare among terrestrial vertebrates and long distance seasonal high-latitude migration is only known to occur in the caribou (*Rangifer tarandus*)^92^. In addition to developmental constraints, migration is limited by body size and its positive allometry with locomotory speed and energetic efficiency^1,7,93^. The smallest extant terrestrial vertebrate migrators are small antelopes with an adult body mass of 20 kg^93^. Osteohistological evidence suggests that Australian high-latitude ornithopods shared similar growth dynamics with their more temperate counterparts, such as *Orodromeus*^94^, growing rapidly in their formative years, an adaptation presumed to have improved their chances against predation and environmental pressures^26^. However, at body masses <1 kg, ‘yearling’ ornithopods fall well below this threshold (Tables 2 and 3). Small-bodied Australian ornithopods appear to have grown at a moderate rate and attained skeletal maturity between five to seven years and maximum body masses of approximately 20 kg (Table 3)^25^. This small body size and relatively low growth rate make seasonal migration an unlikely overwintering strategy for these small dinosaurs, thus it is most likely that they were obligate high-latitude residents.

## Supplementary information

Supplementary Information
