# Supplementary material for: High-latitude neonate and perinate ornithopods from the mid-Cretaceous of southeastern Australia
Source: Sci Rep. 2019 Dec 20;9:19600. doi: 10.1038/s41598-019-56069-8 (PMC6925213; doi:10.1038/s41598-019-56069-8)
Supplement: Supplementary file 1 — Supplementary Information [file 41598_2019_56069_MOESM1_ESM.zip › Kitchener-et-al_SourceCode.html]

High-latitude neonate and perinate ornithopods from the mid-Cretaceous of southeastern Australia


# High-latitude neonate and perinate ornithopods from the mid-Cretaceous of southeastern Australia

#### Justin L. Kitchener, Nicolás E. Campione, Elizabeth T. Smith, Phil R. Bell

Source code compiled by: Nicolás E. Campione and Justin L. Kitchener

### Libraries

```
library(MASSTIMATE)
library(readxl)
```

### Data

```
# Length Data sheet 1
fdata <- read_xlsx("Kitchener et al_Data.xlsx", sheet = "Sheet1"); class(fdata) <- "data.frame"
# Circumference Data sheet 2
cdata <- read_xlsx("Kitchener et al_Data.xlsx", sheet = "Sheet2"); class(cdata) <- "data.frame"
```

### Analyses

##### Length Estimation

```
# simple linear model
fit <- lm(FL~FAP, data=fdata)
summary(fit)
```

```
## 
## Call:
## lm(formula = FL ~ FAP, data = fdata)
## 
## Residuals:
##     Min      1Q  Median      3Q     Max 
## -13.908  -5.824  -2.345   3.342  21.498 
## 
## Coefficients:
##             Estimate Std. Error t value Pr(>|t|)    
## (Intercept)    3.676      6.082   0.605    0.555    
## FAP            7.980      0.426  18.730 2.61e-11 ***
## ---
## Signif. codes:  0 '***' 0.001 '**' 0.01 '*' 0.05 '.' 0.1 ' ' 1
## 
## Residual standard error: 9.914 on 14 degrees of freedom
##   (6 observations deleted due to missingness)
## Multiple R-squared:  0.9616, Adjusted R-squared:  0.9589 
## F-statistic: 350.8 on 1 and 14 DF,  p-value: 2.612e-11
```

```
shapiro.test(fit$residuals)
```

```
## 
##  Shapiro-Wilk normality test
## 
## data:  fit$residuals
## W = 0.92432, p-value = 0.1979
```

```
hist(fit$residuals)
```

```
# linear prediction
fit.pred <- predict(fit, newdata = data.frame(FAP = fdata$FAP[which(fdata$Locality == 
              "Lightning Ridge")], row.names = fdata$Specimen[which(fdata$Locality == 
              "Lightning Ridge")]), interval = "prediction")
fit.pred
```

```
##                fit       lwr       upr
## LRF 759   36.07506  12.67361  59.47652
## LRF 3330  38.70844  15.41096  62.00592
## F105673  103.42575  81.50319 125.34830
## F127930  127.68476 105.64851 149.72100
```

```
# plot
plot(fdata$FAP, fdata$FL, xlab="Anteroposterior diameter at mid point (mm)", ylab="Length (mm)", pch=19, xlim=c(0, 25), ylim=c(0, 200), main="Linear model")
legend("topleft", legend=c("Linear model", "Confidence interval", "Specimen LRF0759 prediction", "Specimen LRF3330 prediction", "Specimen F105673 prediction", "Specimen F127930 prediction"), 
       col = "black", pt.bg = c("black","black","white","white","white","white"), pch = c(-1,-1,22,23,24,25), lty = c(1,2,3,3,3,3),  cex=0.8, bty ="n")

# values for drawing lines
wx = par("usr")[1:2]
new.x = seq(wx[1],wx[2],len=80)

pred = predict(fit, new=data.frame(FAP=new.x), interval="prediction")

# regression line and confidence
lines(new.x,pred[,"fit"], col="black", lwd=2)
lines(new.x,pred[,"lwr"],lty=2)
lines(new.x,pred[,"upr"],lty=2)

# plot LRF predictions
segments(fdata$FAP[which(fdata$Locality == "Lightning Ridge")], fit.pred[,"lwr"], fdata$FAP[which(fdata$Locality == "Lightning Ridge")], fit.pred[,"upr"], col = par("fg"), lty = 3, lwd = par("lwd"))
points(fdata$FAP[which(fdata$Locality == "Lightning Ridge")], fit.pred[,"fit"], col = "black", bg = "white", pch = c(22, 23, 24, 25))
```

##### Mass Estimation

```
# based on Erickson & Tumanova (2000)
DME <- function(juv_proxy, adu_proxy, adu_mass, scale_fac = 3) {
  j.scale <- juv_proxy^scale_fac
  a.scale <- adu_proxy^scale_fac
  j.prop <- j.scale/a.scale
  j.mass <- adu_mass*j.prop
  return(j.mass)
}
```

```
# based on Ramanujan Formulation
ellipse.circ <- function(a, b, diameters = TRUE) {
#measurements are diameters, and must be halved to get the radius
  if(diameters) {
    a <- a/2
    b <- b/2
  }
  h <- ((a-b)^2)/((a+b)^2)
  circ <- pi*(a+b)*(1+(3*h)/(10+sqrt(4-3*h)))
  return(circ)
}
```

```
# NMV P177935 is the largest femur from Woodward (2018) with an EFS, 
# and is used as the adult proxy for DME
adult <- fdata$FL[which(fdata$Specimen == "NMV P177935")]

circ <- fdata$Circumference[which(fdata$Specimen == "NMV P177935")]

largest <- which.max(fdata$FL)
circ <- fdata$Circumference[largest]

cQE(FC = circ)
```

```
##       log.cQE     cQE lower.cQE upper.cQE
## [1,] 4.464309 29127.9   21662.4   36593.4
```

##### Griman Creek Formation specimen Mass Estimation from plain data

```
#LRF mass estimations from linear regression predictions
dme.lin <- data.frame(DME(juv_proxy = fit.pred[,1], adu_proxy = adult, adu_mass = cQE(FC = circ)[2]), 
                      DME(juv_proxy = fit.pred[,1], adu_proxy = adult, adu_mass = cQE(FC = circ)[3]), 
                      DME(juv_proxy = fit.pred[,1], adu_proxy = adult, adu_mass = cQE(FC = circ)[4]))

colnames(dme.lin) <- c("DME", "Lower", "Upper") #results are in grams
dme.lin
```

##### Eumeralla and Wonthaggi specimen Mass Estimation from measured lengths

```
#VIC mass estimations from measured femur lengths
vic.lengths <- na.omit(data.frame(fdata$FL[which(fdata$Datasource == "This study")], 
               row.names = fdata$Specimen[which(fdata$Datasource == "This study")]))
dme.vic <- data.frame(DME(juv_proxy = vic.lengths, adu_proxy = adult, adu_mass = cQE(FC = circ)[2]), 
                      DME(juv_proxy = vic.lengths, adu_proxy = adult, adu_mass = cQE(FC = circ)[3]), 
                      DME(juv_proxy = vic.lengths, adu_proxy = adult, adu_mass = cQE(FC = circ)[4]))

colnames(dme.vic) <- c("DME", "Lower", "Upper") #results are in grams
dme.vic
```

##### Age Estimation

```
# partition data into useful pieces
# Eumeralla and Wonthaggi femora all together
E_W_circs <- cdata[cdata$Locality != "Lightning Ridge",]

# each femur for which there is a series of multiple CGMs and ages
P221151circs <- cdata[cdata$Specimen == "P221151",]
P186326circs <- cdata[cdata$Specimen == "P186326",]
P177935circs <- cdata[cdata$Specimen == "P177935",]

# the Griman Creek femora
LRFcircs <- cdata[cdata$Locality == "Lightning Ridge",]

# linear model of all points
fCirc <- lm(Age~CGMcirc, data=E_W_circs)
#models for each specimen CGM series
MP221151 <- lm(Age~CGMcirc, data=P221151circs)
MP186326 <- lm(Age~CGMcirc, data=P186326circs)
# points for P177935 appear non-linear, so we'll compare a non-linear function with the linear one
# linear model
MP177935 <- lm(Age~CGMcirc, data=P177935circs)
# non-linear model
NP177935 <- nls(Age~b*CGMcirc^m, data=P177935circs, start = list(b = 1, m = 4)) #a power function

# model summaries
summary(fCirc)
```

```
## 
## Call:
## lm(formula = Age ~ CGMcirc, data = E_W_circs)
## 
## Residuals:
##     Min      1Q  Median      3Q     Max 
## -0.9191 -0.6731 -0.2140  0.5706  1.2695 
## 
## Coefficients:
##             Estimate Std. Error t value Pr(>|t|)    
## (Intercept) -2.08196    0.61560  -3.382   0.0038 ** 
## CGMcirc      0.11477    0.01151   9.973 2.85e-08 ***
## ---
## Signif. codes:  0 '***' 0.001 '**' 0.01 '*' 0.05 '.' 0.1 ' ' 1
## 
## Residual standard error: 0.7792 on 16 degrees of freedom
## Multiple R-squared:  0.8614, Adjusted R-squared:  0.8528 
## F-statistic: 99.46 on 1 and 16 DF,  p-value: 2.852e-08
```

```
summary(MP221151)
```

```
## 
## Call:
## lm(formula = Age ~ CGMcirc, data = P221151circs)
## 
## Residuals:
##         7         8         9        10        11        12 
## -0.083545  0.007446  0.098438  0.007628  0.098620 -0.128587 
## 
## Coefficients:
##              Estimate Std. Error t value Pr(>|t|)    
## (Intercept) -4.370505   0.211713  -20.64 3.25e-05 ***
## CGMcirc      0.181802   0.004843   37.54 3.01e-06 ***
## ---
## Signif. codes:  0 '***' 0.001 '**' 0.01 '*' 0.05 '.' 0.1 ' ' 1
## 
## Residual standard error: 0.1037 on 4 degrees of freedom
## Multiple R-squared:  0.9972, Adjusted R-squared:  0.9965 
## F-statistic:  1409 on 1 and 4 DF,  p-value: 3.007e-06
```

```
summary(MP186326)
```

```
## 
## Call:
## lm(formula = Age ~ CGMcirc, data = P186326circs)
## 
## Residuals:
##       13       14       15       16 
##  0.06460 -0.15352  0.07131  0.01762 
## 
## Coefficients:
##              Estimate Std. Error t value Pr(>|t|)   
## (Intercept) -2.604866   0.407764  -6.388   0.0236 * 
## CGMcirc      0.110738   0.007457  14.851   0.0045 **
## ---
## Signif. codes:  0 '***' 0.001 '**' 0.01 '*' 0.05 '.' 0.1 ' ' 1
## 
## Residual standard error: 0.1287 on 2 degrees of freedom
## Multiple R-squared:  0.991,  Adjusted R-squared:  0.9865 
## F-statistic: 220.6 on 1 and 2 DF,  p-value: 0.004503
```

```
summary(MP177935)
```

```
## 
## Call:
## lm(formula = Age ~ CGMcirc, data = P177935circs)
## 
## Residuals:
##      17      18      19      20      21      22 
##  0.5049 -0.5858 -0.7115 -0.1940  0.3236  0.6627 
## 
## Coefficients:
##             Estimate Std. Error t value Pr(>|t|)   
## (Intercept) -5.22442    1.89721  -2.754  0.05118 . 
## CGMcirc      0.16082    0.02839   5.664  0.00479 **
## ---
## Signif. codes:  0 '***' 0.001 '**' 0.01 '*' 0.05 '.' 0.1 ' ' 1
## 
## Residual standard error: 0.6492 on 4 degrees of freedom
## Multiple R-squared:  0.8891, Adjusted R-squared:  0.8614 
## F-statistic: 32.08 on 1 and 4 DF,  p-value: 0.00479
```

```
summary(MP177935)
```

```
## 
## Call:
## lm(formula = Age ~ CGMcirc, data = P177935circs)
## 
## Residuals:
##      17      18      19      20      21      22 
##  0.5049 -0.5858 -0.7115 -0.1940  0.3236  0.6627 
## 
## Coefficients:
##             Estimate Std. Error t value Pr(>|t|)   
## (Intercept) -5.22442    1.89721  -2.754  0.05118 . 
## CGMcirc      0.16082    0.02839   5.664  0.00479 **
## ---
## Signif. codes:  0 '***' 0.001 '**' 0.01 '*' 0.05 '.' 0.1 ' ' 1
## 
## Residual standard error: 0.6492 on 4 degrees of freedom
## Multiple R-squared:  0.8891, Adjusted R-squared:  0.8614 
## F-statistic: 32.08 on 1 and 4 DF,  p-value: 0.00479
```

```
summary(NP177935)
```

```
## 
## Formula: Age ~ b * CGMcirc^m
## 
## Parameters:
##    Estimate Std. Error t value Pr(>|t|)   
## b 0.0002222  0.0003785   0.587  0.58869   
## m 2.4009336  0.4003787   5.997  0.00389 **
## ---
## Signif. codes:  0 '***' 0.001 '**' 0.01 '*' 0.05 '.' 0.1 ' ' 1
## 
## Residual standard error: 0.5052 on 4 degrees of freedom
## 
## Number of iterations to convergence: 38 
## Achieved convergence tolerance: 3.284e-06
```

```
# comparison between linear and non-linear fit for P177935
models <- list(Linear=MP177935, NonLinear=NP177935)
unlist(lapply(models, AIC)) #Non-linear best fit
```

```
##    Linear NonLinear 
##  15.41031  12.40109
```

```
# create plot
plot(E_W_circs$CGMcirc, E_W_circs$Age, xlab="CGM circumference (mm)", ylab="Age (Years)", col = "black", bg = "yellow", pch=21, xlim=c(0, 80), ylim=c(-2, 8), main="Australian small ornithopod femora")
legend("topleft", legend=c("Femora from Woodward (2018)", "Confidence interval", "CGM series specimen NMV P221151", "CGM series specimen NMV P186326", "CGM series specimen NMV P177935", "Specimen LRF 0759 prediction", "Specimen LRF 3330 prediction", "Specimen F105673 prediction", "Specimen F127930 prediction"), 
       col = c("black","black","black","black","black","black","black","black","black"), pt.bg = c("black","black","blueviolet","orange","cornflowerblue","red","red","red","red"), lty = c(1,2,1,1,1,1,3,3,3,3) , pch = c(21,-1,21,21,21,22,23,24,25), cex = 0.8, bty ="n")

# for drawing lines
wx = par("usr")[1:2]
new.x = seq(wx[1],wx[2],len=80)

# predictions
pred = predict(fCirc, new=data.frame(CGMcirc=new.x), interval="prediction")
PP221151 = predict(MP221151, new=data.frame(CGMcirc=new.x), interval="prediction")
PP186326 = predict(MP186326, new=data.frame(CGMcirc=new.x), interval="prediction")
PP177935 = predict(NP177935, new=data.frame(CGMcirc=new.x), interval="prediction")

# lines for each specimen CGM series
lines(new.x,PP221151[,"fit"], col = "blueviolet", lwd = 1, lty = 1)
points(P221151circs$CGMcirc, P221151circs$Age, col = "black", bg = "blueviolet", pch = 21)

lines(new.x,PP186326[,"fit"], col = "orange", lwd = 1, lty = 1)
points(P186326circs$CGMcirc, P186326circs$Age, col = "black", bg = "orange", pch = 21)

lines(new.x,PP177935, col = "cornflowerblue", lwd = 1, lty = 1)
points(P177935circs$CGMcirc, P177935circs$Age, col = "black", bg = "cornflowerblue", pch = 21)

# overall line and confidence
lines(new.x,pred[,"fit"], col="black", lwd=2)
lines(new.x,pred[,"lwr"],lty=2)
lines(new.x,pred[,"upr"],lty=2)

# draw LRF predictions
lrf.pred <- predict(fCirc, newdata = data.frame(CGMcirc = LRFcircs$CGMcirc, row.names = LRFcircs$Specimen), interval = "prediction")

segments(LRFcircs$CGMcirc, lrf.pred[,"lwr"], LRFcircs$CGMcirc, lrf.pred[,"upr"], col = par("fg"), lty = 3, lwd = par("lwd"))
points(LRFcircs$CGMcirc, lrf.pred[,"fit"], col = "black", bg = "red", pch = c(22, 23, 24, 25))
```
